# Femtosecond laser assisted cataract surgery in a cataract patient with a “0 vaulted” ICL: a case report

**DOI:** 10.1186/s12886-020-01440-x

**Published:** 2020-05-05

**Authors:** Yibo Yu, Chengshou Zhang, Yanan Zhu

**Affiliations:** Eye Center of the 2nd Affiliated Hospital, School of Medicine, Zhejiang University, No.88 Jiefang Road, Hangzhou, China

**Keywords:** Femtosecond laser, Cataract surgery, ICL, 0 vault, Case report

## Abstract

**Background:**

Femtosecond laser assisted cataract surgery (FLACS) combined with implantable collamer lenses (ICLs) extraction has been shown to be a feasible method for patients developing cataracts after the ICL implantation. All reported cases had shallow vaults, ranging from 47 μm (μm) to 100 μm. We report for the first time, a case in which the FLACS was performed on the “0” vault eye.

**Case presentation:**

A 38-year-old man with anterior subcapsular cataracts underwent the FLACS combined with ICLs extraction 6 years after ICLs implantation in both eyes. In his left eye, the ICL touched the anterior capsule, existing “0” vault. During the capsulotomy, cavitation bubbles were trapped in the shallow space beneath the ICL, developing from small bubbles into big ones, which resulted in the incomplete capsulotomy. Comparatively, in the right eye, the ICL vault was measured 72 μm, and the capsulotomy was complete and no big cavitation bubbles formed. In both eyes, capsulotomy zones were manually assigned to the anterior capsule surface in the process of laser identification. However, the nuclear pre-fragmentations were unsuccessful in both eyes. Other steps of surgeries were performed uneventfully. Depending on the design of monovision, the uncorrected distance visual acuity (UDVA) was 20/32, and the near uncorrected visual acuity (UCVA) was 20/25 in both eyes postoperatively.

**Conclusions:**

This case suggested that the surgeon should pay attention to the incomplete laser capsulotomy when using a femtosecond laser in cataractous cases with “0” vaulted ICLs, and manual adjustment was required in the process of laser identification.

## Background

The use of femtosecond lasers in cataract surgery has become commonplace, expanding from common cases to complicated cases [1–3]. Special cases have been reported in which the femtosecond laser has been used in cataract patients who have implantable collamer lenses (ICLs) in situ [4, 5]. Combined ICL extraction and femtosecond laser assisted cataract surgery (FLACS) with posterior chamber implantable ocular lens (IOL) implantation has been shown to be a feasible method in these special cases. All reported cases had shallow vaults, ranging from 47 μm (μm) to 100 μm [4, 5].

Here, we discuss a special patient with ICLs who developed cataracts in both eyes, and one of the eyes had a “0” vault. We compare the process of using FLACS between the “0” vault and the shallow vault in one patient and describe the difference in the FLACS in the “0” vault eye.

## Case presentation

A 38-year-old man presented with decreased vision in both eyes for 2 years. The patient had undergone ICL (ICL V4 Visian, STAAR Surgical Co., Monrovia, California, U.S.A.) implantation in both eyes 6 years prior. Postoperatively, the uncorrected distance visual acuity (UDVA) of both eyes was 20/32. The corrected distance visual acuity (CDVA) was similar to the UDVA.

**Fig. 1:**
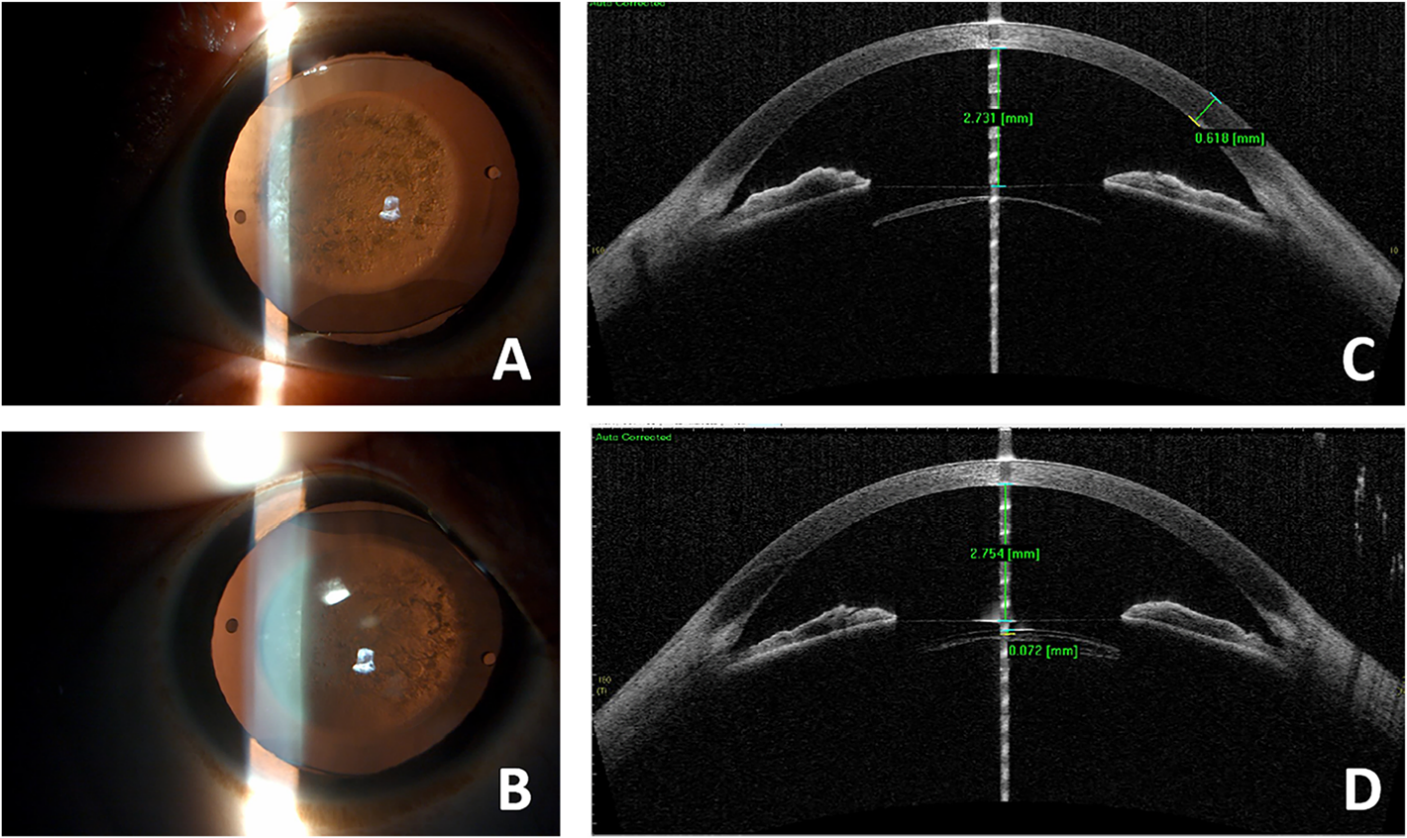
The slit lamp photographs and AS-OCT images of both eyes (**a** to **d**). **a**-**b** The slit lamp photographs showed the lenses’ anterior subcapsular opacities and ICL in situ. **c**-**d** The AS-OCT images of both eyes showed the ICL was touching the anterior capsule of the lens in the left eye (**c**), and the vault in the right eye was 72 μm (**d**)

On examination, ICLs were in situ with anterior subcapsular cataracts in both eyes (Fig. 1). Both eyes underwent anterior segment optical coherence tomography (AS-OCT; CASIA SS-1000, Tomey Corp., Nagoya, Japan) imagining in the model of 3D. Then the ICL vault was manually measured from the back surface of the ICL to the front surface of crystalline lens, centered on the optic axis (shown as a white beam). The vault was shallow in the right eye and measured 72 μm through AS-OCT images. However, in the left eye, the ICL touched the anterior capsule, existing “0” vault (Fig. 1). The endothelial cell density, calculated by the noncontact autofocus specular microscope (EM-3000, Tomey Corp., Nagoya, Japan), was 2587 cells/millimeter squared (mm^2^) in the right eye and 2531 cells/mm^2^ in the left eye.

The patient had planned explantation of the ICL with FLACS after obtaining written informed consent. Considering the unsatisfactory CDVA of the patient after ICL implantation, the surgeon suggested a monovision design for the patient with the implantation of monofocal IOLs rather than multifocal IOLs. And the LenSx laser system (LenSx Laser, Alcon Laboratories, Inc., Fort Worth, Texas, U.S.A.) was used for capsulotomy (5.1 mm diameter, 8 μJ energy) and chop nuclear pre-fragmentation (5.0 mm diameter, 6 chops, 8 μJ energy).

**Fig. 2:**
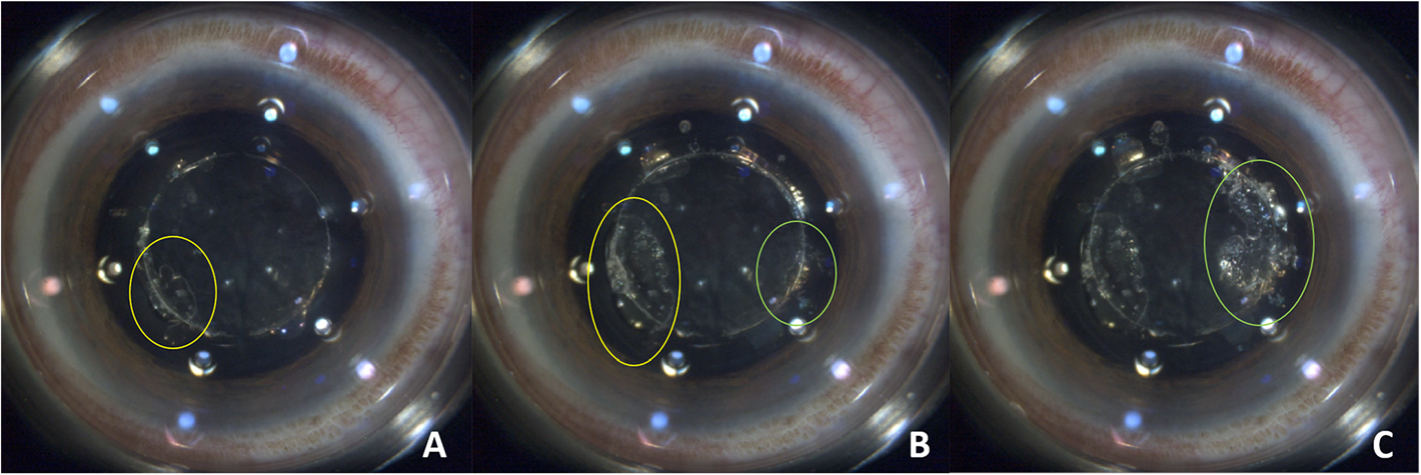
The process of capsulotomy by femtosecond laser in the left eye (**a** to **c**). The yellow circles indicate the big bubble’s formation at two o’clock (from **a** to **b**), while the green circles indicate the big bubble’s formation at nine to 10 o’clock (from **b** to **c**)

The surgery was performed first in the left “0” vault eye. Cavitation bubbles were trapped in the shallow space beneath the ICL around the capsulotomy area during the capsulotomy, developing from small bubbles into big ones (Fig. 2). No additional cavitation bubbles appeared during nuclear pre-fragmentation.

**Fig. 3:**
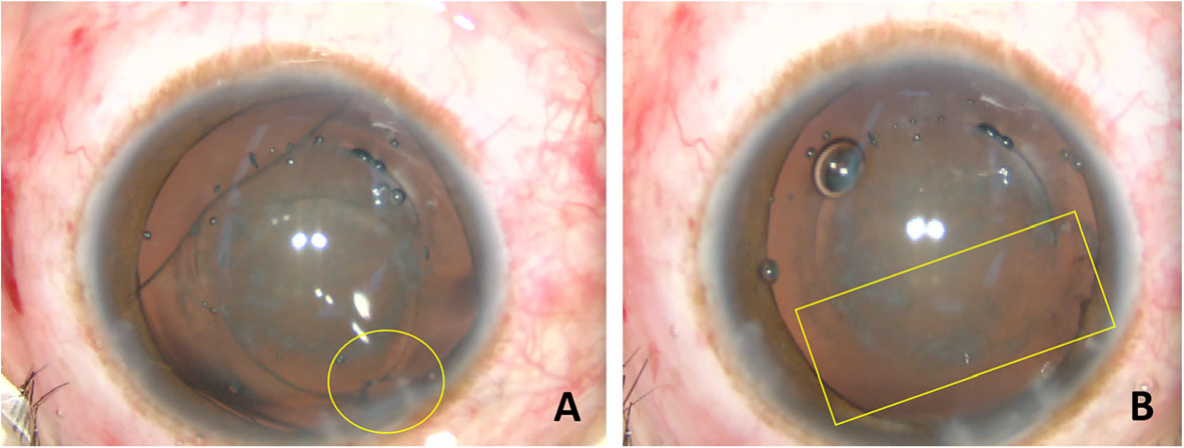
The photographs of the left eye during the surgery (**a** to **b**). **a** The yellow circle shows the rupture of the ICL. **b** The yellow rectangle shows the incomplete area of femtosecond laser assisted capsulotomy

Then, a 2.0 mm primary superior corneal incision was made with a keratome at 135 degrees (°). The sodium hyaluronate 1.7% ophthalmic viscosurgical device (OVD, Amvisc Plus, Bausch & Lomb, Inc.) was injected into the anterior chamber. At first, we attempted to remove the ICL directly without rotating it but failed with the rupture of the ICL. Next, we carefully rotated the ICL. After its vertical angle faced the incision, the ICL was grasped with forceps and extracted through the corneal incision. After the removal of the ICL, the capsulotomy was found to be incomplete between the coordinates of nine o’clock and two o’clock. A second capsulorhexis according to the laser tracks was safely made (Fig. 3). The nuclear pre-fragmentation was unsuccessful and could not be tracked.

The phacoemulsification was manipulated in a standard stop-and-chop manner with the Stellaris system (Bausch & Lomb Laboratories, Rochester, New York, U.S.A.), followed by the implantation of the hydrophobic IOL (Tecnis ZCB00, Abbott Medical Optics Inc., Santa Ana, CA) in the capsular bag.

**Fig. 4:**
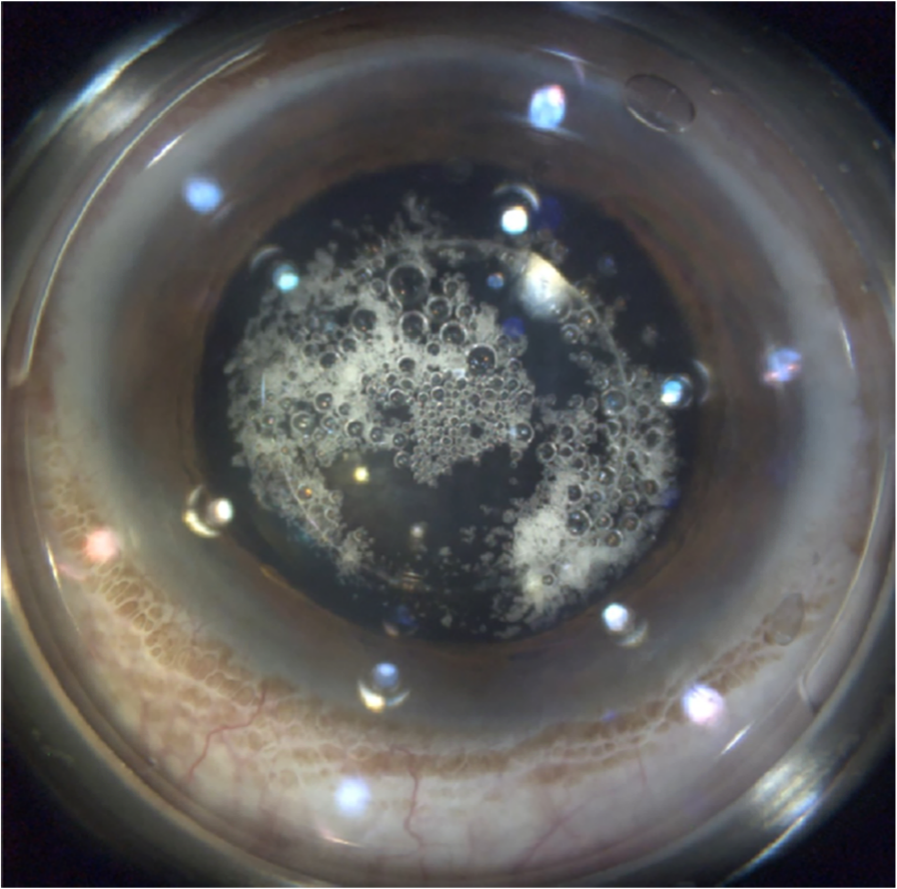
The laser assisted capsulotomy in the right eye. Small cavitation bubbles smoothly dispersed to the central area

The surgery was performed on the right eye one and a half months later. Learning from the experience of the left eye surgery, we made a 3.0 mm temporal corneal incision in the right eye, and the ICL was extracted smoothly without rotation. This time, the capsulotomy was complete. Lots of small cavitation bubbles appeared, dispersing to the central area, and no big bubbles formed (Fig. 4). The nuclear pre-fragmentation, however, failed again. Other steps of the surgery were the same as in the previous surgery and proceeded uneventfully. Both IOLs were well centered in the capsular bag at the end of the surgery.

**Fig. 5:**
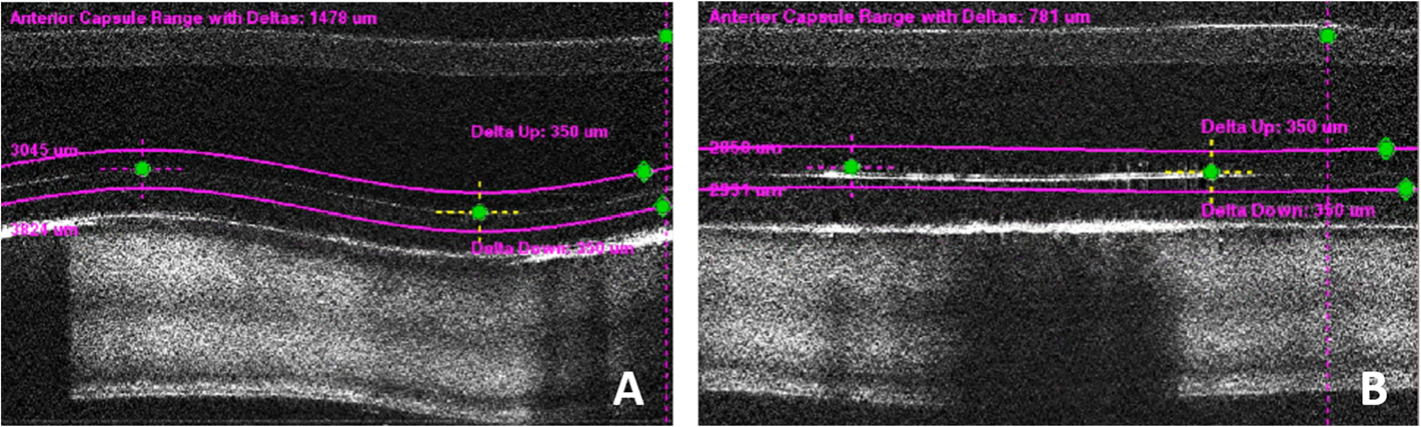
Real-time OCT during FLACS (**a** to **b**). The anterior ICL surfaces were identified as the anterior capsule in both eyes (**a** right eye; **b** left eye)

However, it is worth noting that during the process of laser identification, the anterior ICL surface was accidently confused with the anterior capsule, not only in the shallow vaulted right eye, but also in the “0” vaulted left eye. Manual adjustment by the surgeon was needed to assign treatment zones to the anterior capsule surface in both eyes (Fig. 5).

The patient was instructed to apply topical dexamethasone tobramycin for 2 weeks and pranoprofen for 1 month postoperatively. At the two-week follow-up for the right eye (two-month follow-up for the left eye), in both eyes, the UDVA was 20/32, and the near uncorrected visual acuity (UCVA) was 20/25.

## Discussion and conclusions

Cataracts are the most frequent complication after ICL implantation. The incidence of lens opacification has been reported in the literature as follows: 3 % of eyes at 1 year [6], 4 % to 11% at 2 years [7, 8], 7 % to 13% at 5 years [9, 10], 20% at 8 years [11], and 28% to 58.4% at 10 years [12, 13].

In our case, this patient received the ICL implantation 6 years ago, and developed cataracts almost 2 years ago. A shallow vault was found in both of his eyes. In the right eye, the vault was 72 μm. In the left eye, the AS-OCT showed the posterior surface of the ICL almost touching the anterior lens capsule, an existing “0” vault. The previous literature reported that the mean vault in eyes that developed cataracts was 103 ± 69 μm [14]. The vault in the left eye of our case was lower than the range which is reported.

Surgery is the only option for treating cataracts. The rates of phacoemulsification after ICL implantation are reported as 2 % to 4.9% at 5 years [9, 13], and 17% to 18.7% at 10 years [12, 13]. Our case had cataract surgery 6 years after ICL implantation. Compared with conventional phacoemulsification surgery, the FLACS is a safer and more precise surgery with advantages including more accurate capsulotomy, less corneal endothelial cell loss as well as better and faster visual rehabilitation [1, 2, 15]. Parkhurst et al. and Li S. et al. indicated the efficacy and safety of FLACS in cataract patients with ICLs [4, 5]. However, more cases with different ICL vaults deserve further investigation. With the patient’s strong will to undergo the FLACS, we chose the FLACS for him. Our case is reported as being the first in which the FLACS was performed on the “0” vault eye, and also the first case in which the surgery was performed on both eyes of one patient.

In the “0” vault left eye, we found that the cavitation bubbles were trapped in the shallow space between the ICL and the anterior lens capsule around the capsulotomy area, and that they could not disperse from the capsulotomy area to the center or the peripheral area. During the capsulotomy, small bubbles assembled to become big bubbles. These big bubbles have the potential to grind against the anterior capsule and lead to the position change of the capsule, which is the suggested cause of incomplete capsulotomy. In our case, a second capsulorhexis according to the laser tracks was safely made, which resulted in a well-centered IOL in the capsular bag. Comparatively, in the right eye, because of the remaining vault of 72 μm, the small cavitation bubbles smoothly dispersed to the center of the capsule, and no big bubbles formed; therefore, the complete capsulotomy was achieved. An incomplete capsulotomy was much harder to avoid in the “0” vault eye. We suggested that careful detection and an experienced second capsulorhexis were needed when facing the incomplete capsulotomy. In addition, similar to the shallowly vaulted eye which was reported by Li et al. [4], in our “0” vault eye, manual adjustment of the anterior capsule was also required to achieve laser manipulation correctly in the safe zone.

In our case, unsuccessful nuclear pre-fragmentation happened in both eyes. Parkhurst attributed the incomplete nuclear fragmentation to the frothy cavitation bubbles, which might have interfered with the subsequent laser delivery [5]. However, in the “0” vault eye, although there was no bubble beneath the ICL in the central area to interfere with the laser delivery, pre-fragmentation could not be tracked in the lens either. In our case, we suggested that the anterior subcapsular white opacity of the lens was the main cause of unsuccessful nuclear pre-fragmentation. The white opacity of the lens prevented the laser delivery; therefore, the laser could not make any meaningful cuts in the lens.

Previous literature suggested extracting the ICL through a temporal incision which is identical to the original one [16, 17]. In the left eye of our case, in order to be consistent with regular phacoemulsification in Chinese surgical practices, we made the 2 mm superior incision; however, it was hard to extract the ICL directly. We needed to rotate the ICL to make its vertical angle face the incision, and then we pulled it hard. In the right eye of our case, we chose the 3 mm temporal incision and extracted the ICL directly. By comparison, we agreed with the previous reports, and suggested the temporal incision was more suitable for the ICL extraction.

Our report confirmed the feasibility of using a femtosecond laser in cataractous cases with shallowly vaulted ICLs. However, in the “0” vault eye, because there was little space for cavitation bubbles, the bubbles had the potential to press the anterior capsule downward and change its position. The surgeon should pay close attention to the incomplete laser capsulotomy. Manual adjustment was also required in the process of laser identification in the “0” vault eye.

## Data Availability

All data generated and analyzed during this study are included in this published article.

## Abbreviations

FLACS: Femtosecond laser assisted cataract surgery
ICLs: Implantable collamer lenses
IOL: Implantable ocular lens
UDVA: Uncorrected distance visual acuity
CDVA: Corrected distance visual acuity
UCVA: Uncorrected visual acuity
AS-OCT: Anterior segment optical coherence tomography
OVD: Ophthalmic viscosurgical device
